# HDAC6 regulates NF-κB signalling to control chondrocyte IL-1-induced MMP and inflammatory gene expression

**DOI:** 10.1038/s41598-022-10518-z

**Published:** 2022-04-22

**Authors:** Matt J. Barter, Andrew Butcher, Hui Wang, Dimitra Tsompani, Martin Galler, Ellen L. Rumsby, Kirsty L. Culley, Ian M. Clark, David A. Young

**Affiliations:** 1 Biosciences Institute, Central Parkway, Newcastle University, Newcastle upon Tyne, NE1 3BZ UK; 2 Department of Musculoskeletal and Ageing Science, Institute of Life Course and Medical Sciences, University of Liverpool, Liverpool, UK; 3 Arthritis and Regenerative Medicine Laboratory, Aberdeen Centre for Arthritis and Musculoskeletal Health, University of Aberdeen, Aberdeen, UK; 4 GSK Medicines Research Centre, Gunnels Wood Road, Stevenage, Hertfordshire, SG1 2NY UK; 5 Northern Care Alliance NHS Foundation Trust, Mayo Building, Salford Royal, Stott Lane, Salford, M6 8HD UK; 6 Anglia Innovation Partnership LLP, Centrum, Norwich Research Park, Norwich, UK; 7 School of Biological Sciences, University of East Anglia, Norwich, UK

**Keywords:** Osteoarthritis, Epigenetics, Acetylation, Gene regulation

## Abstract

Elevated pro-inflammatory signalling coupled with catabolic metalloproteinase expression is a common feature of arthritis, leading to cartilage damage, deterioration of the joint architecture and the associated pain and immobility. Countering these processes, histone deacetylase inhibitors (HDACi) have been shown to suppress matrix metalloproteinase (MMP) expression, block cytokine-induced signalling and reduce the cartilage degradation in animal models of the arthritis. In order to establish which specific HDACs account for these chondro-protective effects an HDAC1-11 RNAi screen was performed. HDAC6 was required for both the interleukin (IL)-1 induction of MMP expression and pro-inflammatory interleukin expression in chondrocytes, implicating an effect on NF-κB signalling. Depletion of HDAC6 post-transcriptionally up-regulated inhibitor of κB (IκB), prevented the nuclear translocation of NF-κB subunits and down-regulated NF-κB reporter activation. The pharmacological inhibition of HDAC6 reduced MMP expression in chondrocytes and cartilage collagen release. This work highlights the important role of HDAC6 in pro-inflammatory signalling and metalloproteinase gene expression, and identifies a part for HDAC6 in the NF-κB signalling pathway. By confirming the protection of cartilage this work supports the inhibition of HDAC6 as a possible therapeutic strategy in arthritis.

## Introduction

Cartilage destruction is the predominant characteristic of the arthritides leading to significant joint debilitation^1^. Hyaline cartilage lines the ends of the long bones in articulating joints to provide strength against compressive forces. Chondrocytes constitute the sole cartilage cell type and are responsible for synthesis of all the cartilage ECM macromolecules which provide the tensile strength and water absorptive properties^2,3^. Cartilage damage is largely caused by the action of metalloproteinases, derived from both chondrocytes and synovial cells in rheumatoid arthritis (RA), and predominantly chondrocytes in osteoarthritis (OA). In particular, the matrix metalloproteinase (MMPs) are collectively capable of degrading all components of the cartilage ECM^4^.

Expression of metalloproteinases is tightly regulated during homeostasis but can become dysregulated during disease, in particular in response to proinflammatory cytokines^1^. Inflammation occurs in both the RA and OA joint with inflammatory mediators being released from both invading immune cells and resident cell types. Proinflammatory cytokines, in particular interleukin(IL)-1 and tumour necrosis factor(TNF)-α, but also other interleukin family members such as IL-17 and IL-6, are key mediators in directing the extensive destruction to the cartilage structure^2^. The transcriptional induction of *MMP* expression by these cytokines is well established. Activation of NF-κB and MAPK pathways initiates NF-κB and AP-1 transcription factor transactivation of *MMP* expression and feed-forward loop proinflammatory cytokine expression^5,6^. The NF-κB family of transcription factors includes p65/RelA, RelB, c-Rel, p50 and p52. Inhibitors of NF-κB (IκB) proteins bind to NF-κB in the cytoplasm but upon activation of IκB kinases (IKKs) by proinflammatory signals IκBs are phosphorylated and degraded freeing NF-κB to translocate into the nucleus and transactivate gene expression^7^.

We, and others, have previously implicated a role for histone deacetylation in the regulation of *MMP* expression by proinflammatory cytokines^8–10^. Histones, which constitute the protein content of chromatin, undergo post-translational modifications to regulate their interaction with DNA and control gene expression^11,12^. Acetylation of histones is regulated by the competing influence of histone acetyl transferases and histone deacetylases (HDAC). Broad spectrum HDAC inhibitors (HDACi) completely abrogate the induction of MMPs in chondrocytes thereby preventing ex vivo cartilage degradation^9^. In vivo HDACi treatment of animals undergoing experimental arthritis reduces joint damage and inflammation^8,13,14^. The broad spectrum HDACi used in these studies inhibit most HDACs, but more specific HDACi indicate a role for Class I HDACs in IL-1-induced *MMP* expression and cartilage degradation^8^. HDAC-specific HDACi continue to be developed and together with RNAi-based strategies can be used to determine the specific HDACs involved in *MMP* expression in cartilage^15,16^. The use of such inhibitors may supplement strategies for modulating *MMP* expression to prevent cartilage damage in RA and OA.

HDAC6 is a member of the Class IIb HDAC family which, in contrast to most HDACs, is predominantly localised to the cytoplasm^17^. This is consistent with its major role as a tubulin deacetylase in the control of microtubule dynamics required for cell motility^18^. HDAC6 also has a ubiquitin-binding domain which allows it to determine the fate of ubiquitinated proteins by either preventing their recognition by the ubiquitin and proteasome system (UPS) or for transport of ubiquitinated proteins to aggresomes for autophagic degradation^19^. Consistent with the nuclear role of other HDACs, HDAC6 also has roles in the nucleus regulating transcription, where it can interact with transcription factors and co-repressors, such as RUNX2, NF-κB and LCoR, to direct the repression of target genes^17^.

Recently inhibition of HDAC6 with Tubastatin A has been reported to reduce cartilage damage in experimental OA^20,21^. A role has also been proposed for HDAC6 in inflammatory gene expression and signalling, whereby HDAC6-sepecific inhibitors can reduce the levels of IL-6 in both serum and the paws of collagen-induced arthritic mice^22,23^. Herein we have performed an RNAi screen to identify which specific HDACs are required for *MMP* expression. We identify a prominent role for HDAC6 and explore the mechanism of regulation of *MMP* expression in response to proinflammatory cytokines.

## Results

### HDAC6 is required for IL-1-induced *MMP* expression

**Figure 1:**
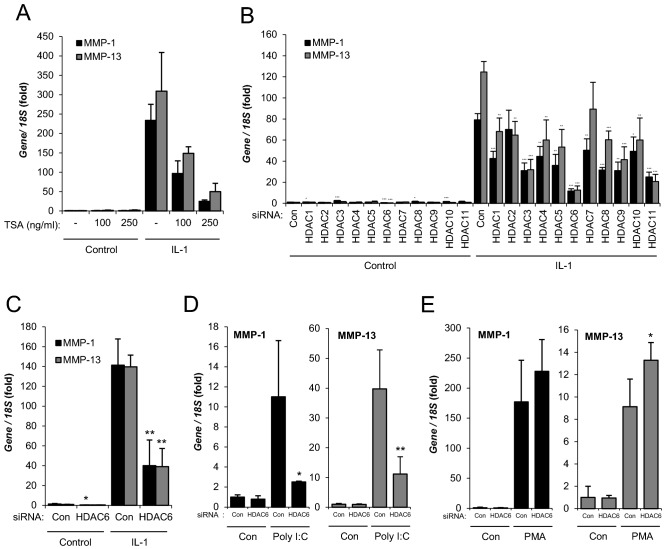
Effect of HDAC6 depletion on *MMP* gene expression in SW1353 cells. (**A**) SW1353 cells were stimulated with IL-1 for 8 h in the presence of TSA at the indicated concentrations. RNA was extracted and expression of *MMP1* and *MMP13* measured by real-time RT-PCR normalised to *18S*. (**B**) SW1353 cells were transfected with the indicated HDAC-targetting siRNAs for 24 h prior to stimulation with IL-1 for 8 h. (**C**–**E**) SW1353 cells were transfected with Dharmacon HDAC6-targetting siRNA for 24 h prior to stimulation with (**C**) IL-1, (**D**) Poly I:C or (**E**) PMA for 8 h. A was performed in duplicate and **B**–**E** were performed in quadruplicate. Data are presented as fold induction relative to the basal expression and represent mean ± S.D. (* *p* < 0.05, ** *p* < 0.01, *** *p* < 0.001). Data are representative of a minimum of three independent experiments.

The induction of *MMP* expression in chondrocytes by pro-inflammatory cytokines such as IL-1 is well documented^1,24^. IL-1-induced expression of *MMP1* and *MMP13* in the human chondrocyte cell line SW1353 was suppressed by the addition of HDAC inhibitor TSA (Fig. 1A) as has been shown previously for a number of HDAC inhibitors^8,9^. A Zn^2+^-dependent class I, II and IV HDAC RNAi screen was performed in order to determine which HDAC family members were required for *MMP* expression (Supplementary Fig. 1). Depletion of a number of HDACs, but especially HDAC3, HDAC6 and HDAC11, reduced the induction of *MMP* expression, particularly for *MMP13* (Fig. 1B). Depletion of HDAC6 showed the greatest repression of both *MMP1* and *MMP13* mRNA levels, indicating the critical requirement for HDAC6 in IL-1-induced *MMP* expression. The effect of TSA and HDAC6 siRNA treatment on histone and tubulin acetylation was confirmed in Supplementary Fig. 1. The role of HDAC6 in *MMP* expression was further validated with an alternative HDAC6-targeting siRNA (Fig. 1C). To examine whether HDAC6 is required for the induction of *MMP* expression by other established inducers of MMPs, cells were stimulated with poly(I:C) and PMA^25,26^. HDAC6 was also required for poly(I:C)-induced *MMP* expression (Fig. 1D), mediated by toll-like receptor(TLR)-activated NF-κB signalling, however, HDAC6 was not required for PMA-induced *MMP* expression, which requires MAPK signalling (Fig. 1E).

### HDAC6 regulates a subset of IL-1-induced genes in SW1353

**Figure 2:**
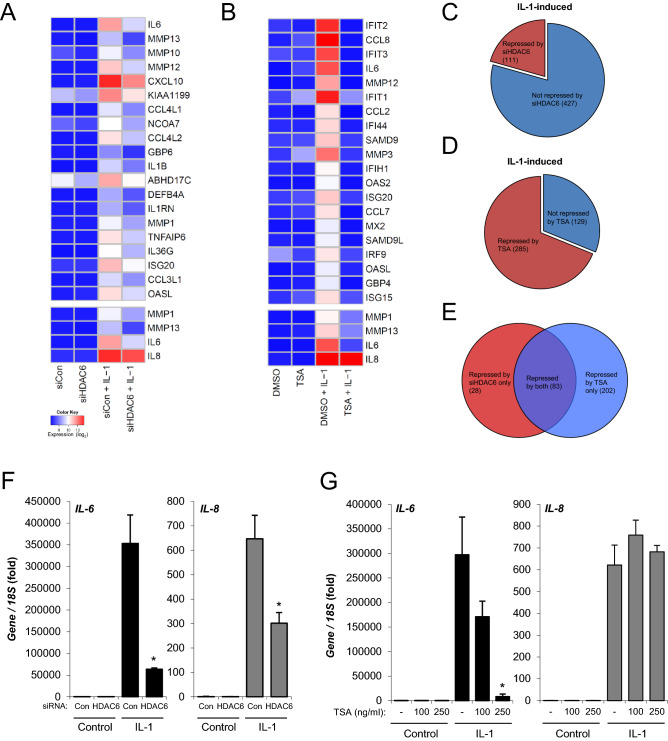
Effect of HDAC6 depletion and TSA on gene expression in SW1353 cells. SW1353 cells were stimulated with IL-1 for 8 h in the presence of HDAC6 siRNA or TSA (250 ng/ml). (**A**–**E**) RNA was extracted and gene expression profiled by whole-genome microarray. (**A**,**B**) Heatmap representation of the IL-1 induced genes most repressed by (**A**) HDAC6 depletion in duplicate or (**B**) TSA treatment in singlicate. The expression of *MMP1*, *MMP13*, *IL6* and *IL8* is provided below each heatmap for comparison. C-E. Venn diagrams representing the proportion of IL-1-induced genes (> 1.5-fold) repressed 1.5-fold by (**C**) HDAC6 depletion, (**D**) TSA treatment, or (**E**) both HDAC6 depletion and TSA. (**F,G**). SW1353 cells were stimulated with IL-1 for 8 h in the presence of (**F**) HDAC6 siRNA or (**G**) TSA at the indicated concentrations. RNA was extracted and expression of *IL6* and *IL8* measured by real-time RT-PCR. D-E were performed in triplicate. Data are presented as fold induction relative to the basal expression and represent mean ± S.D. (*p < 0.05). Data are representative of a minimum of three independent experiments.

To discover the spectrum of chondrocyte genes regulated by HDAC6 Illumina whole genome microarrays were performed following HDAC6 RNAi in combination with IL-1 stimulation (Fig. 2A,B, Supplementary Dataset 1–2). Depletion of HDAC6 reduced the expression of ~ 21% of IL-1-induced genes (Fig. 2C). *MMP13* and *MMP1* were found to be two of the genes most susceptible to experimental knockdown of HDAC6 (Fig. 2A). Specifically *MMP13* was the second most-downregulated IL-1-induced transcript following HDAC6 depletion, second only to *IL6*, another key mediator of cartilage destruction. We also assessed the effect of TSA on whole genome expression in combination with IL-1. Treatment with TSA repressed a greater number of IL-1-induced genes, ~ 70%, than specific depletion of HDAC6 (Fig. 2D). A large proportion of the IL-1-induced genes repressed by depletion of HDAC6 are also suppressed by TSA treatment (Fig. 2E). Of particular note the classically NF-κB-dependent IL-1-induced genes *IL6* and *IL8*^27,28^ are significantly repressed following HDAC6 depletion (Fig. 2A). Interestingly, *IL8* was unaffected by TSA suggesting a deacetylase-independent mechanism of action for HDAC6 (Fig. 2B). Owing to the limited replicates analysed by microarray these results were recapitulated in the independent assessment of *IL6 and IL8* gene expression by real-time PCR following depletion of HDAC6 and inhibition by TSA (Fig. 2F,G).

### HDAC6 is required for NF-κB pathway activation

**Figure 3:**
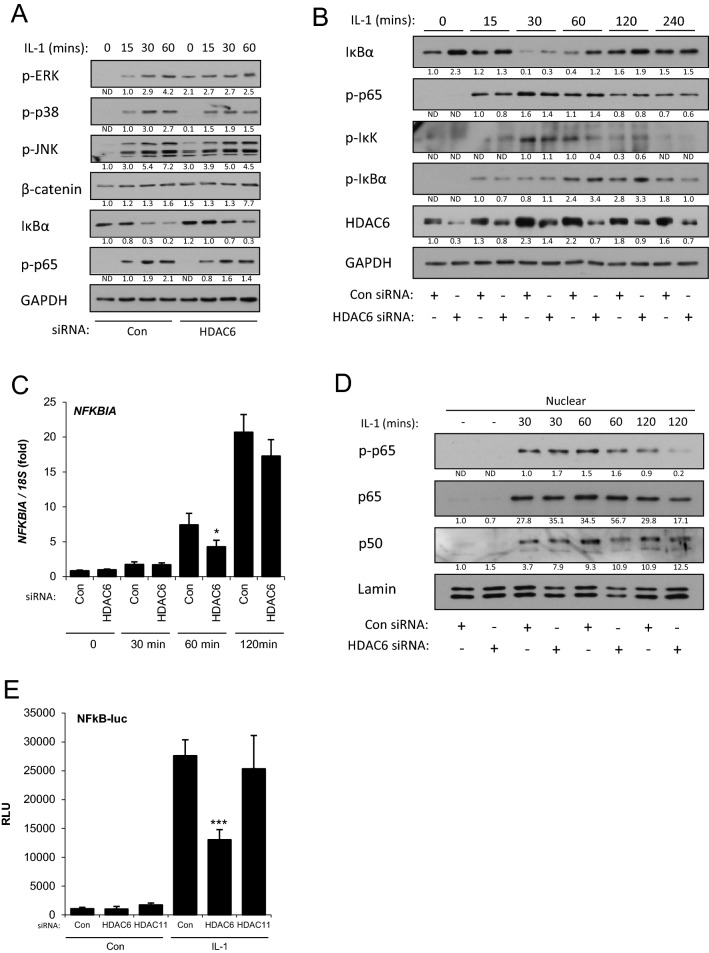
Effect of HDAC6 depletion on NF-κB pathway signalling in SW1353 cells. (**A**–**D**): SW1353 cells were stimulated with IL-1 for the indicated times following transfection with HDAC6-targetting siRNA or control non-targetting siRNA. (**A**–**B**). Total protein was extracted and the activation or abundance of the indicated (**A**) cell signalling pathway or (**B**) NF-κB pathway proteins detected by immunoblotting. HDAC6 levels were measured to confirm knockdown and GAPDH was used to confirm equal protein loading. Densitometric quantification data are shown below each blot as fold change in relation to the first detectable sample, normalised to GAPDH. (**C**) RNA was extracted and expression of NFKBIA (IkBα) measured by real-time RT-PCR. (**D**) Nuclear protein was extracted and the level of the indicated NF-κB pathway proteins detected by immunoblotting. Nuclear Lamin was used to confirm equal protein loading. Densitometric quantification data are shown below each blot as fold change in relation to the first detectable sample, normalised to Lamin (**E**). Luciferase expression in SW1353 cells stimulated with IL-1 for 24 h following transfection with HDAC6 or HDAC11-targetting siRNA or control non-targetting siRNA and an NF-κB luciferase reporter. Values are the mean ± SD, * = *P* < 0.05, *** = *P* < 0.001 versus non-targetting control. Data are representative of a minimum of three independent experiments.

IL-1 receptor binding activates a cascade of signalling pathways in particular NF-κB and MAPK^29^. In addition HDAC6 may regulate Wnt signalling through enhancing β-catenin nuclear translocation^30,31^. Accordingly, the activation of intracellular signalling pathways by IL-1 was assessed following HDAC6 RNAi. HDAC6 knockdown had no effect on the activation of MAPK pathways ERK, p38 or JNK, nor WNT signalling, indicated by β-catenin degradation (Fig. 3A). However, the steady state levels of IκBα increased following HDAC6 depletion and the extent of degradation was reduced. A more detailed time-course of NF-κB pathway activation confirmed the increase in basal IκBα levels following HDAC6 knockdown (Fig. 3B). Levels of IkBα also returned to pre-stimulation levels more rapidly. IκBα is induced by NF-κB to elicit its negative feedback activity, however, the phosphorylation of p65 (p-p65) was unaffected indicating no increased activation of pathway signalling at that level. In addition, the levels of IκBα transcript (*NFKBIA*) were not regulated by HDAC6 depletion suggesting the effect of HDAC6 was post-transcriptional (Fig. 3C). A reduction in IKK phosphorylation was found, partially consistent with the timing of IκBα degradation, but not with the initial upregulation of IκBα. To determine whether decreased HDAC6 and the increased levels of IκBα were functionally impacting upon the NF-κB pathway we assessed the nuclear translocation of NF-κB subunits. After over 30 min of IL-1 stimulation, HDAC6 depletion reduced the levels of both phosphorylated p65 as a proportion of total p65 in the nucleus (Fig. 3D). In addition, the expression of an NF-κB-dependent luciferase reporter construct following IL-1 stimulation was reduced in HDAC6-depleted cells (Fig. 3E).

### HDAC6 is essential for *MMP* and interleukin expression and NF-κB signalling in human articular chondrocytes

**Figure 4:**
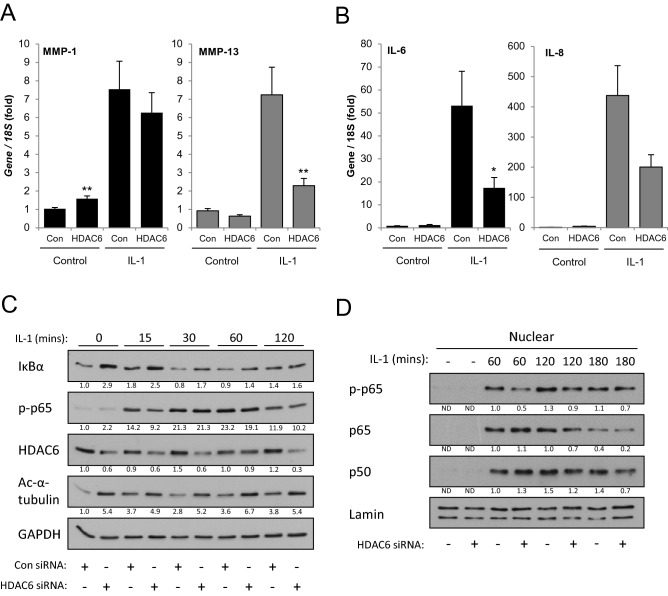
Effect of HDAC6 depletion on *MMP* gene expression and NF-κB pathway signalling in HAC. (**A**–**B**) HAC cells were transfected with Dharmacon HDAC6-targetting siRNA for 24 h prior to stimulation with IL-1 for 8 h. RNA was extracted and expression of (**A**) *MMP1* and *MMP13*, (**B**) *IL6* and *IL8* measured by real-time RT-PCR. Data are presented as fold induction relative to the basal expression and represent mean of quadruplicate samples ± S.D. (*p < 0.05, **p < 0.01, ***p < 0.001). Data are representative of a minimum of three independent experiments. (**C–D**) HAC cells were stimulated with IL-1 for the indicated times following transfection with HDAC6-targetting siRNA or control non-targetting siRNA. (**C**) Total protein was extracted and the activation or abundance of the indicated NF-κB pathway proteins detected by immunoblotting. HDAC6 levels were measured to confirm knockdown and GAPDH was used to confirm equal protein loading. (**D**) Nuclear protein was extracted and the level of the indicated NF-κB pathway proteins detected by immunoblotting. Nuclear Lamin was used to confirm equal protein loading. (**C**–**D**). Densitometric quantification data are shown below each blot as fold change in relation to the first detectable sample, normalised to (**C**) GAPDH or (**D**) Lamin. Data are representative of a minimum of three independent experiments.

To confirm biological relevance the role of HDAC6 in IL-1-induced gene expression was examined in primary chondrocytes (HAC). HDAC6 was again found to be essential for maximal induction of *MMP13* expression in HAC by IL-1 stimulation, but there was no significant effect on *MMP1* levels (Fig. 4A). Furthermore, the induction of the proinflammatory cytokines *IL6* and *IL8* was also repressed by HDAC6 depletion (Fig. 4B). Accounting for these observations the levels and localisation of NF-κB signalling components was again dysregulated by depletion of HDAC6. IκBα levels were increased at the basal level following HDAC6 depletion and following IL-1 stimulation the extent of IκBα degradation was reduced (Fig. 4C). The IL-1-induced nuclear localisation of NF-κB subunits was also dependent on HDAC6 (Fig. 4D), and taken together recapitulated the role of HDAC6 in SW1353 cells. The functional impact of HDAC6 depletion on tubulin acetylation was also confirmed (Fig. 4C).

### HDAC6 inhibitors repress *MMP* expression and cartilage collagen release

**Figure 5:**
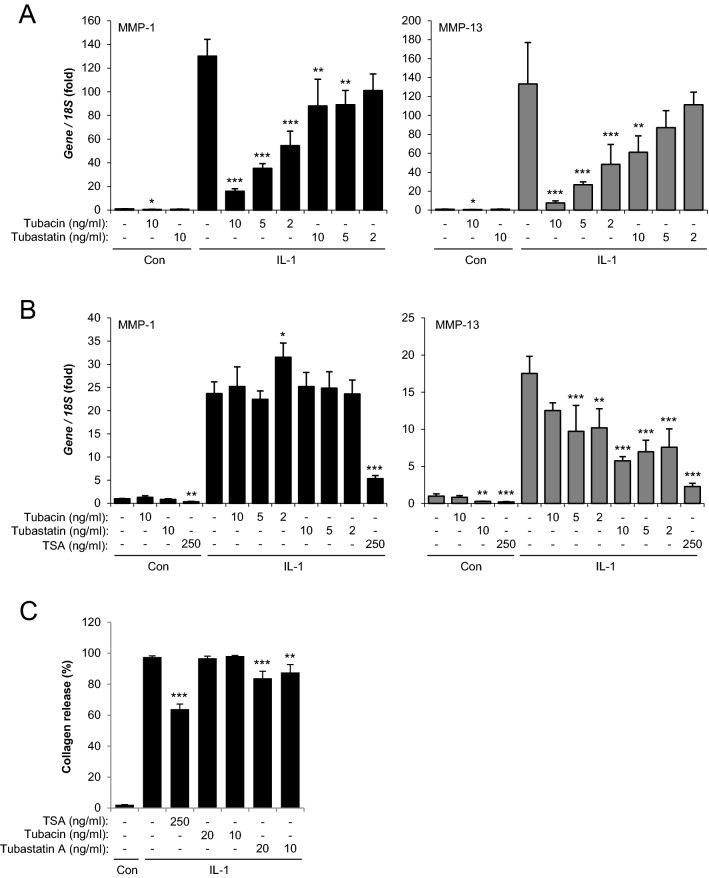
Effect of HDAC6 inhibition on *MMP* expression and cartilage degradation. A–B. (**A**) SW1353 and (**B**) HAC cells were stimulated with IL-1 for (**A**) 8 or (**B**) 24 h in the presence of HDACi at the indicated concentrations. RNA was extracted and expression of *MMP1* and *MMP13* measured by real-time RT-PCR normalised to *18S*. Data are presented as fold induction relative to the basal expression and represent mean ± S.D. (**C**) Bovine nasal cartilage was cultured in serum-free medium in the presence of either medium alone, or medium containing IL-1 and HDACi TSA, tubacin, tubasatin A or DMSO control (0.01% v/v) at the indicated concentrations for 14 days. The levels of collagen fragments released into the medium were determined by measurement of hydroxyproline after day 14 of culture and expressed as a percentage of the total (mean ± S.D). All experiments were performed in quadruplicate. Significance was analysed compared to IL-1 alone, where, *p < 0.05, **p < 0.01, ***p < 0.001. Data are representative of two independent experiments.

The above data support both deacetylase-dependent and -independent mechanisms by HDAC6. To further investigate the role of HDAC6 in IL-1-induced chondrocyte gene expression the effect of pharmacological inhibitors of HDAC6 deacetylase activity was assessed. *MMP* induction in SW1353 cells was significantly repressed by Tubacin and to a lesser extent Tubastatin A (Fig. 5A), two selective HDAC6 inhibitors. In HAC again only IL-1-induced *MMP13* expression was repressed by the HDAC6 inhibitors (Fig. 5B), in contrast with the inhibition of both *MMP1* and *MMP13* by pan-HDAC inhibitor TSA. To assess the functional impact of reduced *MMP* expression and proinflammatory signalling following HDAC6 depletion we examined the effect of HDAC6 inhibitors on the degradation of bovine nasal cartilage explants catalysed by IL-1. Treatment with HDAC6 inhibitor Tubastatin A significantly reduced the extent of collagen release after 14 days of stimulation indicating the role of HDAC6 in cartilage turnover (Fig. 5C).

## Discussion

Disease progression in osteoarthritis remains a largely intractable condition where the joint damage epitomised by cartilage destruction continues to develop, resulting in significant morbidity. Experimental models of arthritis have proven amenable to treatment with a number of pharmacological agents, including HDACi. Initially HDACi, especially TSA, were demonstrated to reduce joint damage in inflammatory models of arthritis, concomitant with a reduction in synovitis grade and cytokine levels^14,32,33^. We have demonstrated that TSA also reduces joint damage in an experimental OA model involving destabilization of the medial meniscus (DMM) in mice^8^ consistent with the effect of HDACi treatment in a rabbit anterior cruciate ligament transection (ACLT) model^34^. This builds on previous studies demonstrating in vitro and ex vivo ability of HDACi to prevent cartilage resorption and inhibit pro-inflammatory cytokine-induced catabolic gene expression^9,10,35^. Consequently this positions HDACs as a possible therapeutic target in the arthritides.

TSA is a broad spectrum HDACi which selectively inhibits class I, II and IV Zn^2+^-dependent class HDACs^36^. However long-term treatment of chronic, non–life-threatening diseases such as OA with broad-spectrum HDACi is not appropriate due to their toxicity. We have now comprehensively depleted cells specifically of each HDAC to determine which may account for the ability of TSA to significantly block *MMP* expression. Consistent with our findings^8^ depletion of class I HDACs, HDAC1, 3 and 8, significantly reduced *MMP* expression. Taken together this reduction could account for a large part of the induction of *MMP* expression by IL-1, in line with the abrogation of *MMP* expression by class I selective HDACi MS-275. Considering this we were surprised to note that depletion of HDAC6 elicited the most significant reduction of IL-1-induced *MMP* expression. Remarkably the induction of *MMP13* by IL-1 was the second most dependent upon the presence of HDAC6, after *IL6*. MMP13 has been proposed as the critical collagenase in OA collagen degradation as opposed to MMP1 in RA^1^. Thus the potential to downregulate MMP13 levels effectively would offer substantial therapeutic benefit for OA.

The effect of HDAC6 depletion was reiterated following stimulation by poly I:C which, like IL-1, also initiates NF-κB signalling pathway activation^25^, whereas the PMA induction of *MMP* expression was unaffected. Phorbol esters, such as PMA, activate protein kinase C (PKC) which predominantly signals through the MAPK pathways^37^. This suggests a susceptibility in NF-κB signalling, activated via a TRAF6 intermediate by both the IL-1 receptor and TLR3, in response to IL-1 and poly I:C respectively, rather than a MAPK pathway mechanism. We also observed that HDAC6 depletion of IL-1 stimulated cells did not affect activation of MAPK signalling pathways.

TSA treatment or HDAC6 depletion identified a substantial proportion of NFκB-dependent genes whose expression was susceptible to both HDAC inhibition and HDAC6 loss. The requirement for NF-κB in the IL-1 induction of *MMP* expression in chondrocytes is well established^38–42^. In the context of in vivo experimental animal models, the inhibition of NF-κB pathway signalling can suppress catabolic gene expression, including MMPs, and limit osteoarthritis development^43,44^. HDACi have previously been demonstrated to suppress the NF-κB pathway in chondrocytes. Resveratrol, a pan HDACi, blocks NF-κB signalling in chondrocytes by suppressing IL-1-induced IκBα degradation^45^. Vorinostat, inhibitor of class I and II HDACs, blocks IL-1 induction of MMPs by inhibiting p38, ERK and NF-κB pathway signalling, specifically by blocking NF-κB translocation to the nucleus in chondrocytes^46^. Our data implies that HDAC6 may in part account for the effect of these HDACi. Interestingly, class III HDACs the Sirtuins, may have an opposing role in the NF-κB pathway, by directly deacetylating NF-κB p65 to reduce its transcriptional activity^47^. In particular, in chondrocytes SIRT1 activation inhibited the activation of NF-κB and inflammatory gene expression^48,49^.

NF-κB is a transcriptional regulator of IL-1-induced genes in both SW1353 cells and HAC^50^. Two particularly important proinflammatory cytokines in arthritis are IL-6 and IL-8, both activated by NF-κB-dependent mechanisms^27,28^. Herein *IL6* and *IL8* were significantly repressed following HDAC6 depletion, but only *IL6* by HDAC inhibition. IL-6 is fundamental in RA-driven cartilage destruction both in activation of immune cells but also induction of degradative enzymes such as the MMPs^51^. Blockade of IL-6 signalling with tocilizumab, an antibody against the IL-6 receptor, is an approved treatment in RA^52^. Cytokine-mediated inflammation has an increasingly recognised contribution to the development of OA^1^. Although the level of inflammation compared to RA appears low the combinatorial effect of IL-1, IL-6 and IL-8 amongst other cytokines may support MMP induction and precipitate cartilage destruction. In chondrocytes IL-6 induces *MMP* expression and blockade of IL-6 and its signalling proved efficacious in treating experimental OA in mice^53^. Similarly IL-6 neutralisation blocked HIF2α-induced cartilage destruction and MMP induction in mice, although *IL6-/-* mice developed more advanced osteoarthritis upon aging^54,55^. NF-κB also potently induces HIF-2α which is required for the development of OA in mice^56^.

The basal and post-stimulus recovery levels of IκBα were elevated following HDAC6 depletion. IκBα is an inherently unstable protein and as such is continuously synthesised, the majority forming a stable complex with NF-κB^57^. The stimulus-induced degradation of IκBα is initiated by phosphorylation by IKK and leads to ubiquitination of IκBα N-terminal lysine residues by β-TrCP E3 ubiquitin ligase and degradation by the UPS^58^. Interestingly HDAC6 inhibitors can increase N-terminus lysine acetylation of the Wnt signalling pathway mediator β-catenin resulting in its reduced ubiquitination by β-TrCP^59^. However, there are no reports of acetylation of IκB itself. VCP/p97, which facilitates delivery of ubiquitinated proteins to the UPS, has been demonstrated to mediate cytokine-induced degradation of IκBα^60^. HDAC6 is known to form a complex with VCP/p97 but it remains to be determined whether HDAC6 directly influences VCP/p97-mediated IκBα degradation^17,61^. Interestingly, a recent study using HDAC6 inhibitor ACY-1215 in IL-1-treated chondrocytes also showed reduced *MMP* expression, reduced activation of NF-κB signalling and elevated IκBα levels post-stimulation, although the basal levels of IκBα were unaffected in contrast to the effect of HDAC6 depletion herein^62^.

HDAC6 also ensures efficient delivery of ubiquitinated substrates to the autophagic machinery for degradation and is known to interact with key autophagy chaperone HSC70^63,64^. The deacetylase domain-independent binding of VCP/p97, ubiquitin or other unknown substrates by HDAC6 may account for the genes regulated by HDAC6 depletion but not by TSA treatment, including *IL8*. Stress, or heat shock proteins (HSPs), which are also induced by cytokines, act as chaperones to confer stability on proteins. HDAC6 deacetylates some HSPs, to regulate their chaperone activity, and also controls HSP expression via transcription factor HSF1^65–68^. Importantly HSP90, HSP70 and HSP27 can positively/negatively regulate NF-κB signalling^69–72^. Oxidative stress due to accumulation of reactive oxygen species (ROS) induces autophagy and HDAC6 is also linked directly to redox regulation through deacetylation of Prx proteins thereby limiting their H_2_0_2_ reduction activity^73^.

Herein inhibition of HDAC6 with Tubastatin A blocked cartilage degradation in line with the studies identifying protection against cartilage damage with Tubastatin A treatment in the DMM model of experimental OA^20,21^. Tubastatin A was developed to address issues with the high lipophilicity of Tubacin, which may account for Tubacin’s lack of effect in our 14 day cartilage degradation model^74^. Both experimental OA studies infer the protection offered by Tubastatin A is associated with the role of HDAC6 in autophagy and ROS regulation. Shen et al. showed that Tubastatin A treatment of chondrocytes and mice activated autophagy and increased cell viability while reducing cartilage degradation^20^. Zheng et al. suggest that Tubastatin A disrupts regulation of mitochondrial connectivity and function by HDAC6 leading to ROS reduction and reduced cartilage damage^21^.

Elsewhere acetylation plays a key role in the NF-κB pathway. NF-κB proteins regulate transcription in part through recruitment of HATs and HDACs to promoters^75^. In fact IκBα has also been documented to interact with HDACs 1, 3 and 5 to regulate gene expression, but again no IκBα acetylation was found^75^. Acetylation of the NF-κB proteins themselves is well documented. NF-κB p50 and p65 are acetylated by p300, each at a number of lysine residues, which may promote DNA binding or influence association with IκBa, although the exact effect differs between studies^75^. HDAC3 and SIRT1 are able to deacetylate NF-κB p65^75^. Additionally, HDAC3, by removing inhibitory NF-κB p65 lysine acetylation, is able to promote the transcription of IL-1-induced genes^76^. A further study found the acetylation of NF-κB p65 increased in HDAC3-deficient chondrocytes but this conversely led to the activation of NF-κB^77^. A more recent study also suggests that HDAC6 could deacetylate NF-κB p65 to reduce its DNA-binding activity^78^. However, such a mechanism is converse to the loss of NF-κB signalling-dependent gene activation which we see following depletion of HDAC6. The primary cilia also positively regulates IL-1 signalling to NF-κB in chondrocytes^79^. HDAC6 can deacetylate and destabilise cilia microtubules leading to cilia disassembly. Accordingly, inhibition of HDAC6 should maintain cilia function and allow NF-κB pathway activation, in contrast to the findings herein.

Inhibition of NF-κB activation is a key therapeutic goal for autoimmune diseases and a number of cancers. Upregulation of IκB levels has been attempted by a number of mechanisms including blocking of IκB ubiquitination with E3 ligase inhibition, stabilisation of IκB by engineering a dominant repressor or using decoy cell-penetrating IκB phosphopeptides to impair recruitment of β-TrCP to IκB^58^. Our data indicates that inhibition/depletion of HDAC6 may also represent a strategy to increase IκB levels and downregulate NF-κB pathway signalling.

## Conclusion

Interestingly HDAC6 function appears intricately linked with chondrocyte biology. Mutation in the HDAC6 3’UTR abolishes a microRNA binding site causing upregulation of HDAC6 is linked to a form of X-linked chondrodysplasia, suggesting a specific role for HDAC6 in chondrocytes^80^. Additionally, HDAC6 KO mice have increased tibial bone mineral density^81^, and loss or inhibition of HDAC6 can cause an increase in growth plate proliferation and ossified bone in mouse model of Thanatophoric Dysplasia Type II (TDII)^82^.

The work herein highlights that HDAC6 also has an important role in chondrocyte pro-inflammatory signalling and metalloproteinase gene expression. It establishes the hitherto unrecognised function of HDAC6 in NF-κB signalling and validates that specific inhibition of HDAC6 can impact upon cartilage degradation. With the ongoing development of specific HDACi this work supports the inhibition of HDAC6 as a possible therapeutic strategy in the arthritides.

## Methods

### Human cells and cartilage treatment

Human articular chondrocytes (HACs) were derived from articular cartilage obtained from consenting patients following hip or knee replacement surgery with Ethical Committee approval from the Newcastle and North Tyneside Health Authority (UK) for all experimental protocols. Informed consent was obtained from all subjects and all methods were carried out in accordance with relevant guidelines and regulations. Enzymatic digestion of tissue and maintenance and culture of cells were as previously described^83^. Cells were used at passage 1, where passage 0 corresponds to the cells released from the cartilage by enzymatic digestion. Human chondrosarcoma cells (SW1353) were cultured in Dulbecco's modified Eagle's medium supplemented with 10% fetal bovine serum, 2 mM/l-glutamine, 100 IU/ml penicillin, and 100 μg/ml streptomycin. Cells were seeded 1 day before treatment and cultured overnight in serum-free medium, prior to stimulation. Recombinant human IL-1α was a gift from Dr Keith Ray (Glaxo-SmithKline, Stevenage, UK). Recombinant human OSM was donated by Professor John Heath (Department of Biochemistry, University of Birmingham, Edgbaston, UK). HMW poly(I-C) (Invivogen, Toulouse, France). Phorbol 12-myristate 13-acetate (PMA), Tubacin and Tubastatin A were purchased from Sigma-Aldrich (Poole, UK), while TSA was from Calbiochem (Nottingham, UK). A CytoTox-Glo Cytotoxicity Assay was performed on SW1353 cells treated with a range of TSA concentrations for 6 h following manufacturer’s instructions (Supplementary Fig. 2).

### RNAi transfection

Cells plated overnight were transfected at 50% confluence with 50 nM (in SW1353) or 100 nM (in HAC) siRNA using Dharmafect 1 lipid reagent (Horizon Discovery, Cambridge, UK) according to the manufacturer's protocol and essentially as described previously^83^. FlexiTube siRNAs used were as follows: Hs_HDAC1_6, Hs_HDAC2_1, Hs_HDAC2_2, Hs_HDAC3_1, Hs_HDAC3_2, Hs_HDAC4_3, Hs_HDAC4_4, Hs_HDAC5_1, Hs_HDAC5_4, Hs_HDAC6_5, Hs_HDAC7A_6, Hs_HDAC8_2, Hs_HDAC8_4, Hs_HDAC9_1, Hs_HDAC9_3, Hs_HDAC10_1, Hs_HDAC10_2, Hs_HDAC11_4, Hs_HDAC11_6, or non-targeting control siRNA (AllStars Negative Control siRNA; Qiagen, Manchester, UK). Dharmacon siRNA SMARTpools used were as follows: D-001206-14-20 non-targeting siRNA and D-043456-04 (HDAC6) (Horizon Discovery). Following 24 h transfection cells were washed and cultured in serum-free medium overnight before stimulation.

### RNA extraction, real-time RT-PCR and Illumina whole-genome microarray

Total RNA was extracted from cells with Cells-to-cDNA II lysis buffer and cDNA synthesis was performed using MMLV reverse transcriptase and random hexamers according to the manufacturer's protocol (ThermoFisher Scientific, Loughborough, UK). TaqMan or SYBR green RT-PCR were performed and gene expression levels calculated as previously described^84^. Primer sequences are listed in Supplementary Table 1. For microarray total RNA was extracted with RNAeasy kit (Qiagen). Illumina whole-genome expression microarray Human HT-12 V4 (Illumina Inc., Illumina United Kingdom, Saffron Walden, UK) was used to profile gene expression of RNA samples according to the manufacturer's protocol. The HDAC6 siRNA experiment was performed in duplicate treatment with either HDAC6- or negative control siRNA (Qiagen), plus or minus IL-1. The TSA experiment was performed in singlicate with TSA or DMSO treatment, plus or minus IL1. Raw expression data were processed using R/Bioconductor package lumi with a variance stabilising transformation and robust spline normalization as standard^85^. Expression analysis was performed in R/bioconductor limma package by fitting a linear model and applying empirical Bayes smoothing as standard^86^. Where multiple probes detect a single transcript the average expression value was used. Heatmaps were generated with the R gplots package.

### Immunoblotting

Whole cell lysates were prepared using a modified Schindler buffer as described previously^84^. Nuclear extracts were generated using the Nuclear and Cytoplasmic Extraction Reagent kit (ThermoFisher Scientific) according to the manufacturer's protocol. Lysates were resolved by SDS-PAGE electrophoresis, transferred to PVDF membranes (Millipore, Watford, UK) and subsequently probed using the following antibodies: phospho-extracellular signal regulated kinase (ERK)1/2 (phospho-p44/42) (no. 9101), phospho-p38 (no. 9211), phospho-c-Jun N-terminal kinase (JNK) (no. 9251), phospho-Akt (Ser473; no. 9271), nuclear factor κ light polypeptide gene enhancer in B cells inhibitor IκBα (no. 9242), phospho-p65, p65, β-catenin, phospho-IKK, p-IκBα, p50, Lamin, HDAC6, acetyl-α-tubulin, acetyl-Histone H3 from Cell Signaling Technology (Danvers, Massachusetts, USA). A mouse monoclonal anti-glyceraldehyde 3’-phosphate dehydrogenase (GAPDH) antibody (clone 6C5; MAB374) was purchased from Chemicon (Hampshire, UK). The polyclonal secondary immunoglobulins/horseradish peroxidase were from Cytomation (Dako, Glostrup, Denmark). The quantitation of protein levels was performed by densitometric analysis with ImageJ as standard and normalised to housekeeping protein GAPDH or lamin.

### Luciferase assay

SW1353 cells were seeded into 96 well plates at 18,000 cells/cm2. Each well was transfected with 25 ng of NF-kB luciferase reporter (Takara Bio, Saint-Germain-en-Laye, France) along with 1.5 ng Renilla (pRL-TK Vector, Promega, Southampton, UK) control reporter vector using FuGENE HD transfection reagent (Roche, Lewes, UK) as previously described^87^. Transfected cells were serum starved overnight prior to stimulation with IL-1α (0.5 ng/ml) for 6 h. PBS-washed cells were lysed with Passive Lysis buffer and luminescence monitored using a Glomax Luminometer and the Dual-Luciferase Reporter Assay System (Promega). Firefly luciferase data were normalised to the Renilla luciferase control.

### Bovine nasal cartilage assay

Bovine cartilage was dissected from nasal septi obtained from a local abattoir. Bovine nasal septum cartilage was dissected into approximately 2-mm^3^ discs, plated into 24-well tissue culture plates (3 discs per well, n = 4) and cultured in serum-free Dulbecco’s modified Eagle medium as described previously^88^. Fresh serum-free media with/without cytokines and test reagents were then added (day 0). At day 7, culture supernatants were harvested and replaced with fresh medium containing the same test reagents as day 0. Cartilage and culture supernatants were harvested at day 14 and the remaining cartilage was digested with papain. Hydroxyproline release was assayed as a measure of collagen degradation, and the extent of release was calculated as a percentage of the total.

### Statistical analysis

Statistical differences between sample groups were assessed using students t-test for single comparisons or one-way ANOVA for multiple comparisons, where *p < 0.05; **p < 0.01 and ***p < 0.001.

## Supplementary Information

Supplementary Information 1.

Supplementary Figures.

Supplementary Figure 1.

Supplementary Figure 2.

Supplementary Figure 3.

Supplementary Table 1.

Supplementary Information 2.

## Data Availability

SW1353 microarray data is deposited at GEO (GSE186690).

## Abbreviations

HDAC: Histone deacetylase
IL-1: Interleukin-1
NF-κB: Nuclear factor kappa B
OA: Osteoarthritis
MMP: Matrix metalloprotease

## References

[1] Pap T, Korb-Pap A (2015). Cartilage damage in osteoarthritis and rheumatoid arthritis–two unequal siblings. Nat. Rev. Rheumatol..

[2] Goldring MB, Marcu KB (2009). Cartilage homeostasis in health and rheumatic diseases. Arthritis Res. Ther..

[3] Guilak F (2018). Osteoarthritis as a disease of the cartilage pericellular matrix. Matrix Biol..

[4] Rowan AD (2008). Metalloproteases as potential therapeutic targets in arthritis treatment. Expert. Opin. Ther. Targets.

[5] Vincenti MP, Brinckerhoff CE (2002). Transcriptional regulation of collagenase (MMP-1, MMP-13) genes in arthritis: Integration of complex signaling pathways for the recruitment of gene-specific transcription factors. Arthritis Res..

[6] Young, D.A., M.J. Barter, and D.J. Wilkinson, Recent advances in understanding the regulation of metalloproteinases. *F1000Res* 8 (2019). 10.12688/f1000research.17471.1 PMC6381797 30828429

[7] Hinz M, Scheidereit C (2014). The IkappaB kinase complex in NF-kappaB regulation and beyond. EMBO Rep..

[8] Culley KL (2013). Class I histone deacetylase inhibition modulates metalloproteinase expression and blocks cytokine-induced cartilage degradation. Arthritis Rheum..

[9] Young DA (2005). Histone deacetylase inhibitors modulate metalloproteinase gene expression in chondrocytes and block cartilage resorption. Arthritis Res. Ther..

[10] Wang X (2009). Inhibition of histone deacetylases antagonized FGF2 and IL-1beta effects on MMP expression in human articular chondrocytes. Growth Factors.

[11] Bannister AJ, Kouzarides T (2011). Regulation of chromatin by histone modifications. Cell Res..

[12] Venkatesh S, Workman JL (2015). Histone exchange, chromatin structure and the regulation of transcription. Nat. Rev. Mol. Cell Biol..

[13] Nasu Y (2008). Trichostatin A, a histone deacetylase inhibitor, suppresses synovial inflammation and subsequent cartilage destruction in a collagen antibody-induced arthritis mouse model. Osteoarthritis Cartilage.

[14] Nishida K (2004). Histone deacetylase inhibitor suppression of autoantibody-mediated arthritis in mice via regulation of p16INK4a and p21(WAF1/Cip1) expression. Arthritis Rheum..

[15] Falkenberg KJ, Johnstone RW (2014). Histone deacetylases and their inhibitors in cancer, neurological diseases and immune disorders. Nat. Rev. Drug Discov..

[16] Ho TCS, Chan AHY, Ganesan A (2020). Thirty years of HDAC inhibitors: 2020 insight and hindsight. J. Med. Chem..

[17] Li Y, Shin D, Kwon SH (2013). Histone deacetylase 6 plays a role as a distinct regulator of diverse cellular processes. FEBS J..

[18] Hubbert C (2002). HDAC6 is a microtubule-associated deacetylase. Nature.

[19] Boyault C (2007). HDAC6, at the crossroads between cytoskeleton and cell signaling by acetylation and ubiquitination. Oncogene.

[20] Shen Z (2021). Inhibition of HDAC6 by Tubastatin A reduces chondrocyte oxidative stress in chondrocytes and ameliorates mouse osteoarthritis by activating autophagy. Aging (Albany NY).

[21] Zheng Y (2020). Inhibition of histone deacetylase 6 by tubastatin a attenuates the progress of osteoarthritis via improving mitochondrial function. Am. J. Pathol..

[22] Lee J (2015). A novel histone deacetylase 6-selective inhibitor suppresses synovial inflammation and joint destruction in a collagen antibody-induced arthritis mouse model. Int. J. Rheum. Dis..

[23] Vishwakarma S (2013). Tubastatin, a selective histone deacetylase 6 inhibitor shows anti-inflammatory and anti-rheumatic effects. Int. Immunopharmacol..

[24] Chan CM (2017). Cytokine-induced MMP13 expression in human chondrocytes is dependent on activating transcription factor 3 (ATF3) regulation. J. Biol. Chem..

[25] Radwan M (2013). Matrix metalloproteinase 13 expression in response to double-stranded RNA in human chondrocytes. Arthritis Rheum..

[26] Tetlow LC, Adlam DJ, Woolley DE (2001). Matrix metalloproteinase and proinflammatory cytokine production by chondrocytes of human osteoarthritic cartilage: Associations with degenerative changes. Arthritis Rheum..

[27] Mukaida N, Mahe Y, Matsushima K (1990). Cooperative interaction of nuclear factor-kappa B- and cis-regulatory enhancer binding protein-like factor binding elements in activating the interleukin-8 gene by pro-inflammatory cytokines. J. Biol. Chem..

[28] Shimizu H (1990). Involvement of a NF-kappa B-like transcription factor in the activation of the interleukin-6 gene by inflammatory lymphokines. Mol. Cell Biol..

[29] Weber A, Wasiliew P, Kracht M (2010). Interleukin-1 (IL-1) pathway. Sci. Signal.

[30] Li Y (2008). HDAC6 is required for epidermal growth factor-induced beta-catenin nuclear localization. J. Biol. Chem..

[31] Zhu J, Coyne CB, Sarkar SN (2011). PKC alpha regulates Sendai virus-mediated interferon induction through HDAC6 and beta-catenin. EMBO J..

[32] Chung YL (2003). A therapeutic strategy uses histone deacetylase inhibitors to modulate the expression of genes involved in the pathogenesis of rheumatoid arthritis. Mol. Ther..

[33] Lin HS (2007). Anti-rheumatic activities of histone deacetylase (HDAC) inhibitors in vivo in collagen-induced arthritis in rodents. Br. J. Pharmacol..

[34] Chen WP (2010). Alleviation of osteoarthritis by Trichostatin A, a histone deacetylase inhibitor, in experimental osteoarthritis. Mol. Biol. Rep..

[35] Chabane N (2008). Histone deacetylase inhibitors suppress interleukin-1beta-induced nitric oxide and prostaglandin E2 production in human chondrocytes. Osteoarthritis Cartilage.

[36] Khan N (2008). Determination of the class and isoform selectivity of small-molecule histone deacetylase inhibitors. Biochem. J..

[37] Schonwasser DC (1998). Activation of the mitogen-activated protein kinase/extracellular signal-regulated kinase pathway by conventional, novel, and atypical protein kinase C isotypes. Mol. Cell Biol..

[38] Raymond L (2007). RelA is required for IL-1beta stimulation of Matrix Metalloproteinase-1 expression in chondrocytes. Osteoarthritis Cartilage.

[39] Mengshol JA (2000). Interleukin-1 induction of collagenase 3 (matrix metalloproteinase 13) gene expression in chondrocytes requires p38, c-Jun N-terminal kinase, and nuclear factor kappaB: Differential regulation of collagenase 1 and collagenase 3. Arthritis Rheum..

[40] Ma B (2013). T cell factor 4 is a pro-catabolic and apoptotic factor in human articular chondrocytes by potentiating nuclear factor kappaB signaling. J. Biol. Chem..

[41] Liacini A (2002). Inhibition of interleukin-1-stimulated MAP kinases, activating protein-1 (AP-1) and nuclear factor kappa B (NF-kappa B) transcription factors down-regulates matrix metalloproteinase gene expression in articular chondrocytes. Matrix Biol..

[42] Fan Z (2006). Role of mitogen-activated protein kinases and NFkappaB on IL-1beta-induced effects on collagen type II, MMP-1 and 13 mRNA expression in normal articular human chondrocytes. Rheumatol. Int..

[43] Chen LX (2008). Suppression of early experimental osteoarthritis by in vivo delivery of the adenoviral vector-mediated NF-kappaBp65-specific siRNA. Osteoarthritis Cartilage.

[44] Kobayashi H (2016). Biphasic regulation of chondrocytes by Rela through induction of anti-apoptotic and catabolic target genes. Nat. Commun..

[45] Shakibaei M (2008). Resveratrol suppresses interleukin-1beta-induced inflammatory signaling and apoptosis in human articular chondrocytes: Potential for use as a novel nutraceutical for the treatment of osteoarthritis. Biochem. Pharmacol..

[46] Zhong HM (2013). Vorinostat, a HDAC inhibitor, showed anti-osteoarthritic activities through inhibition of iNOS and MMP expression, p38 and ERK phosphorylation and blocking NF-kappaB nuclear translocation. Int. Immunopharmacol..

[47] Yeung F (2004). Modulation of NF-kappaB-dependent transcription and cell survival by the SIRT1 deacetylase. EMBO J.

[48] Moon MH (2013). SIRT1, a class III histone deacetylase, regulates TNF-alpha-induced inflammation in human chondrocytes. Osteoarthritis Cartilage.

[49] Matsushita T (2013). The overexpression of SIRT1 inhibited osteoarthritic gene expression changes induced by interleukin-1beta in human chondrocytes. J Orthop Res.

[50] Gebauer M (2005). Comparison of the chondrosarcoma cell line SW1353 with primary human adult articular chondrocytes with regard to their gene expression profile and reactivity to IL-1beta. Osteoarthritis Cartilage.

[51] Hashizume M, Mihara M (2011). The roles of interleukin-6 in the pathogenesis of rheumatoid arthritis. Arthritis.

[52] Oldfield V, Dhillon S, Plosker GL (2009). Tocilizumab: a review of its use in the management of rheumatoid arthritis. Drugs.

[53] Latourte, A., et al., Systemic inhibition of IL-6/Stat3 signalling protects against experimental osteoarthritis. Ann. Rheum. Dis. (2016). 10.1136/annrheumdis-2016-209757 27789465

[54] Ryu JH (2011). Interleukin-6 plays an essential role in hypoxia-inducible factor 2alpha-induced experimental osteoarthritic cartilage destruction in mice. Arthritis. Rheum..

[55] de Hooge AS (2005). Male IL-6 gene knock out mice developed more advanced osteoarthritis upon aging. Osteoarthritis Cartilage.

[56] Saito T (2010). Transcriptional regulation of endochondral ossification by HIF-2alpha during skeletal growth and osteoarthritis development. Nat. Med..

[57] Mathes E (2008). NF-kappaB dictates the degradation pathway of IkappaBalpha. EMBO J..

[58] Kanarek N (2010). Ubiquitination and degradation of the inhibitors of NF-kappaB. Cold. Spring Harb. Perspect. Biol..

[59] Iaconelli J (2015). HDAC6 inhibitors modulate Lys49 acetylation and membrane localization of beta-catenin in human iPSC-derived neuronal cells. ACS Chem. Biol..

[60] Li JM (2014). The p97-UFD1L-NPL4 protein complex mediates cytokine-induced IkappaBalpha proteolysis. Mol. Cell. Biol..

[61] Boyault C (2006). HDAC6-p97/VCP controlled polyubiquitin chain turnover. EMBO J..

[62] Cheng C (2019). ACY-1215 exhibits anti-inflammatory and chondroprotective effects in human osteoarthritis chondrocytes via inhibition of STAT3 and NF-kappaB signaling pathways. Biomed. Pharmacother..

[63] Pandey UB (2007). HDAC6 rescues neurodegeneration and provides an essential link between autophagy and the UPS. Nature.

[64] Zhang L (2015). Proteomic identification and functional characterization of MYH9, Hsc70, and DNAJA1 as novel substrates of HDAC6 deacetylase activity. Protein Cell.

[65] Boyault C (2007). HDAC6 controls major cell response pathways to cytotoxic accumulation of protein aggregates. Genes Dev.

[66] Gibert B (2012). Knock down of heat shock protein 27 (HspB1) induces degradation of several putative client proteins. PLoS ONE.

[67] Kovacs JJ (2005). HDAC6 regulates Hsp90 acetylation and chaperone-dependent activation of glucocorticoid receptor. Mol. Cell.

[68] Rao R (2008). HDAC6 inhibition enhances 17-AAG–mediated abrogation of hsp90 chaperone function in human leukemia cells. Blood.

[69] Alford KA (2007). Heat shock protein 27 functions in inflammatory gene expression and transforming growth factor-beta-activated kinase-1 (TAK1)-mediated signaling. J. Biol. Chem..

[70] De Nardo D (2005). A central role for the Hsp90.Cdc37 molecular chaperone module in interleukin-1 receptor-associated-kinase-dependent signaling by toll-like receptors. J. Biol. Chem..

[71] Parcellier A (2003). HSP27 is a ubiquitin-binding protein involved in I-kappaBalpha proteasomal degradation. Mol. Cell. Biol..

[72] Ran R (2004). Hsp70 promotes TNF-mediated apoptosis by binding IKK gamma and impairing NF-kappa B survival signaling. Genes Dev..

[73] Parmigiani RB (2008). HDAC6 is a specific deacetylase of peroxiredoxins and is involved in redox regulation. Proc. Natl. Acad. Sci. U S A.

[74] Butler KV (2010). Rational design and simple chemistry yield a superior, neuroprotective HDAC6 inhibitor, tubastatin A. J. Am. Chem. Soc..

[75] Calao M (2008). A pervasive role of histone acetyltransferases and deacetylases in an NF-kappaB-signaling code. Trends Biochem. Sci..

[76] Ziesche E (2013). The coactivator role of histone deacetylase 3 in IL-1-signaling involves deacetylation of p65 NF-kappaB. Nucleic Acids Res..

[77] Carpio LR (2016). Histone deacetylase 3 supports endochondral bone formation by controlling cytokine signaling and matrix remodeling. Sci Signal.

[78] Yang CJ (2015). Nuclear HDAC6 inhibits invasion by suppressing NF-kappaB/MMP2 and is inversely correlated with metastasis of non-small cell lung cancer. Oncotarget.

[79] Wann AK, Chapple JP, Knight MM (2014). The primary cilium influences interleukin-1beta-induced NFkappaB signalling by regulating IKK activity. Cell Signal.

[80] Simon D (2010). A mutation in the 3'-UTR of the HDAC6 gene abolishing the post-transcriptional regulation mediated by hsa-miR-433 is linked to a new form of dominant X-linked chondrodysplasia. Hum. Mol. Genet..

[81] Zhang Y (2008). Mice lacking histone deacetylase 6 have hyperacetylated tubulin but are viable and develop normally. Mol. Cell Biol..

[82] Ota S (2016). HDAC6 deficiency or inhibition blocks FGFR3 accumulation and improves bone growth in a model of achondroplasia. Hum. Mol. Genet..

[83] Zhang Q (2008). Differential Toll-like receptor-dependent collagenase expression in chondrocytes. Ann. Rheum. Dis..

[84] Barter MJ (2010). Lipophilic statins prevent matrix metalloproteinase-mediated cartilage collagen breakdown by inhibiting protein geranylgeranylation. Ann. Rheum. Dis..

[85] Du P, Kibbe WA, Lin SM (2008). lumi: a pipeline for processing Illumina microarray. Bioinformatics.

[86] Smyth GK (2004). Linear models and empirical bayes methods for assessing differential expression in microarray experiments. Stat. Appl. Genet. Mol. Biol..

[87] Barter MJ (2015). Genome-wide MicroRNA and gene analysis of mesenchymal stem cell chondrogenesis identifies an essential role and multiple targets for miR-140-5p. Stem Cells.

[88] Shingleton WD (2000). Retinoic acid combines with interleukin-1 to promote the degradation of collagen from bovine nasal cartilage: Matrix metalloproteinases-1 and -13 are involved in cartilage collagen breakdown. J. Cell Biochem..

