# Supplementary material for: HDAC6 regulates NF-κB signalling to control chondrocyte IL-1-induced MMP and inflammatory gene expression
Source: Sci Rep. 2022 Apr 22;12:6640. doi: 10.1038/s41598-022-10518-z (PMC9033835; doi:10.1038/s41598-022-10518-z)
Supplement: Supplementary file 1 — Supplementary Information 1. [file 41598_2022_10518_MOESM1_ESM.pptx]

## Slide 1
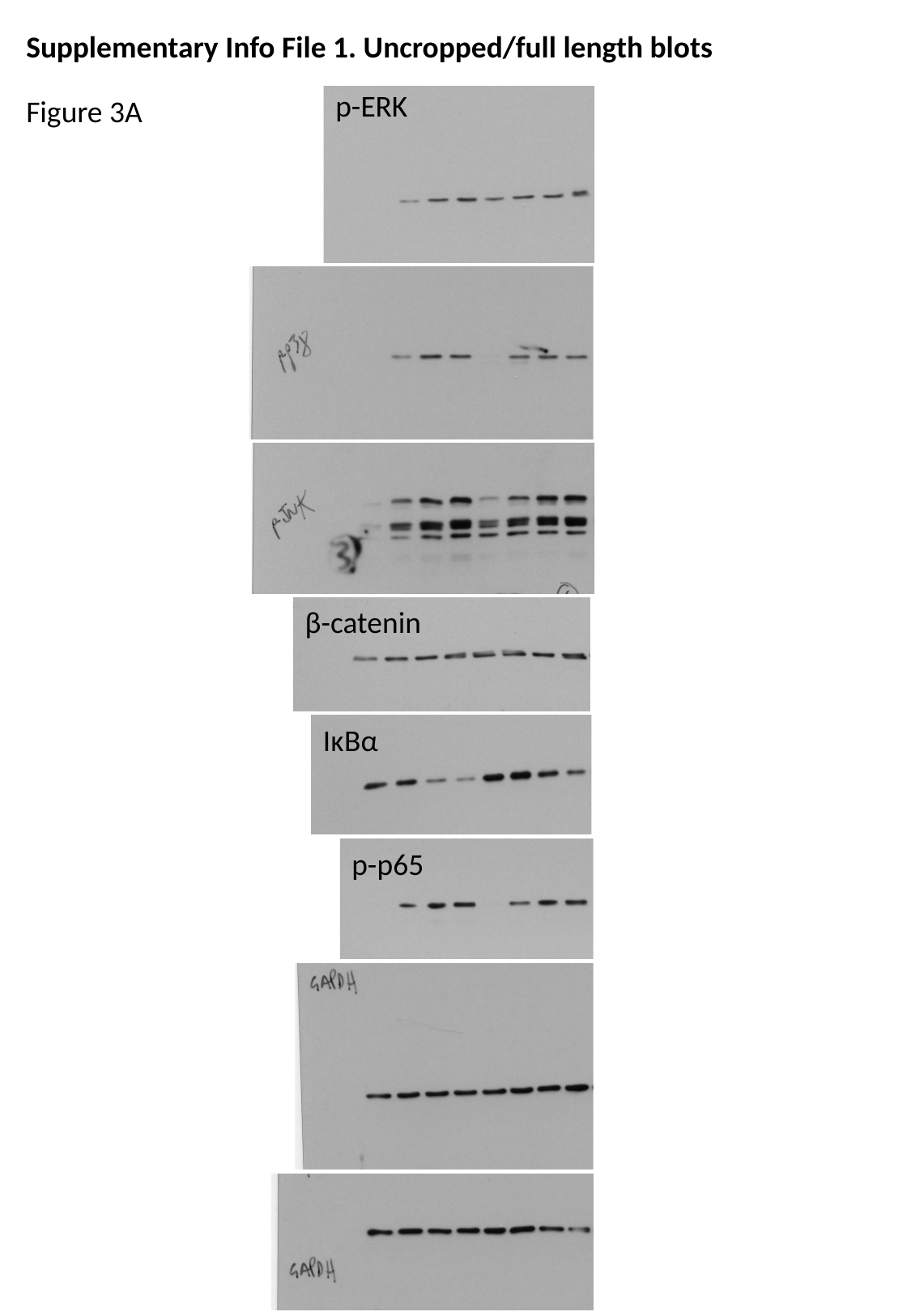

Supplementary Info File 1. Uncropped/full length blots
p-ERK
Figure 3A
β-catenin
IᴋBα
p-p65

## Slide 2
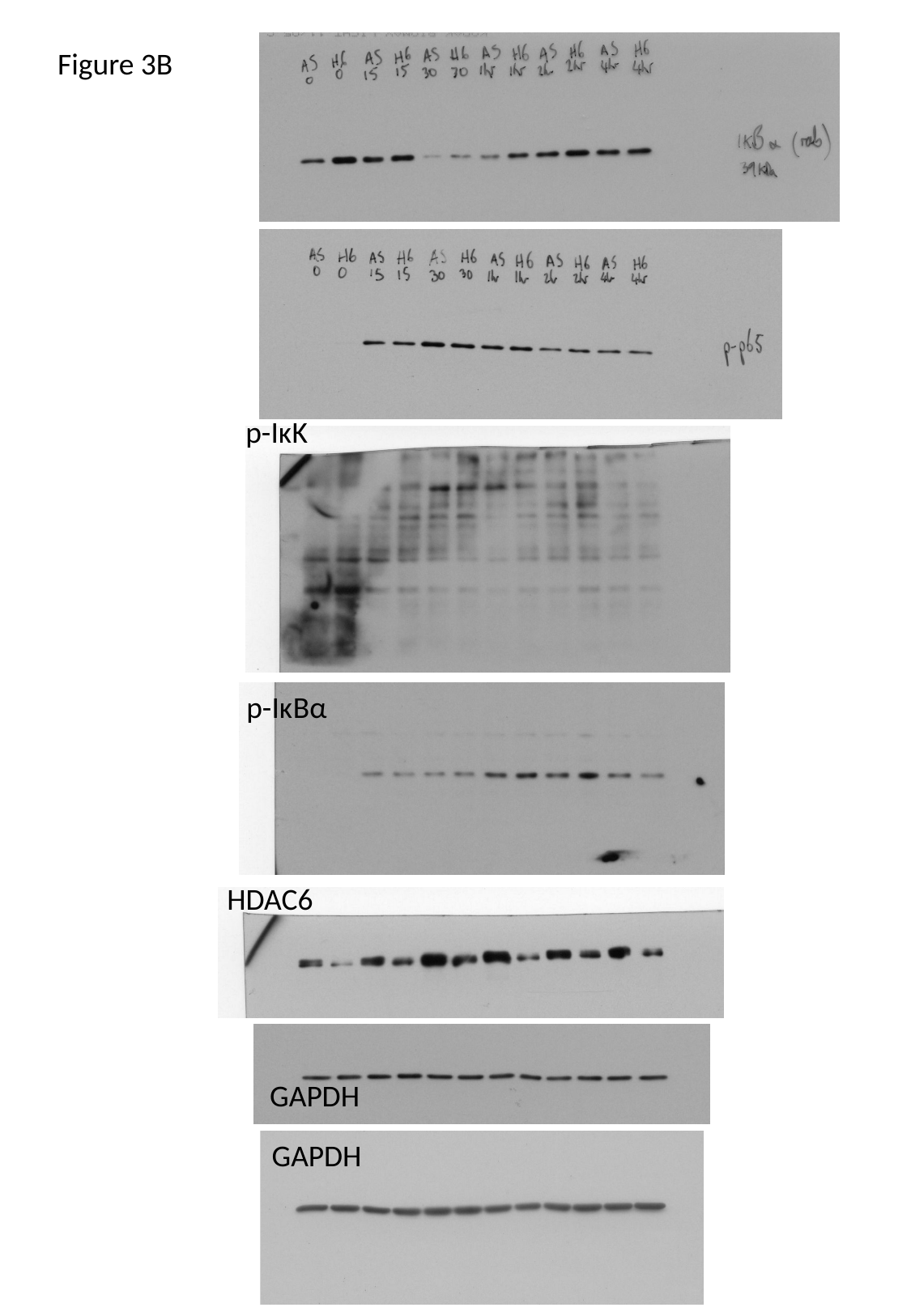

Figure 3B
p-IᴋK
p-IᴋBα
HDAC6
GAPDH
GAPDH

## Slide 3
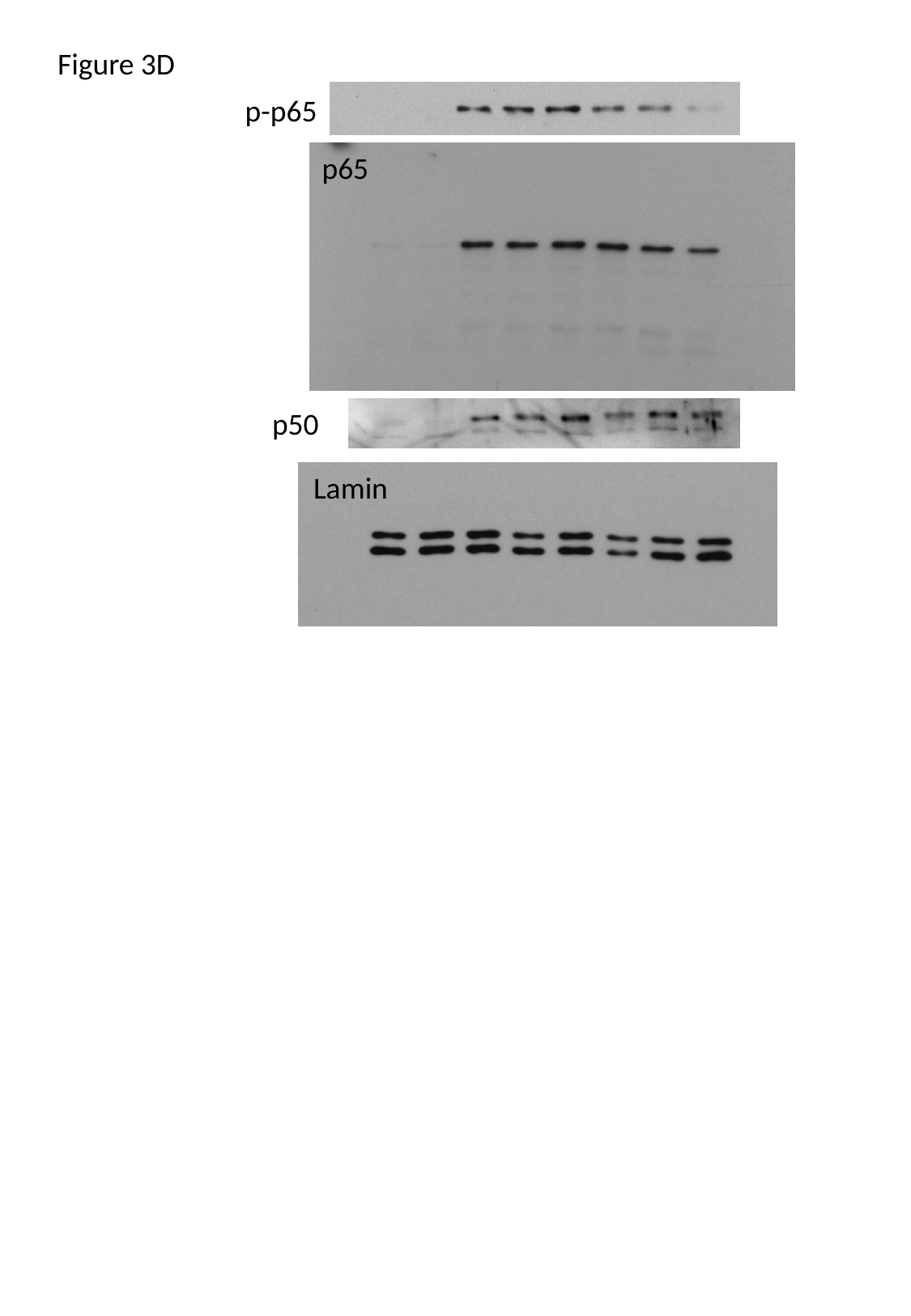

Figure 3D
p-p65
7/7/10
p65
p50
Lamin

## Slide 4
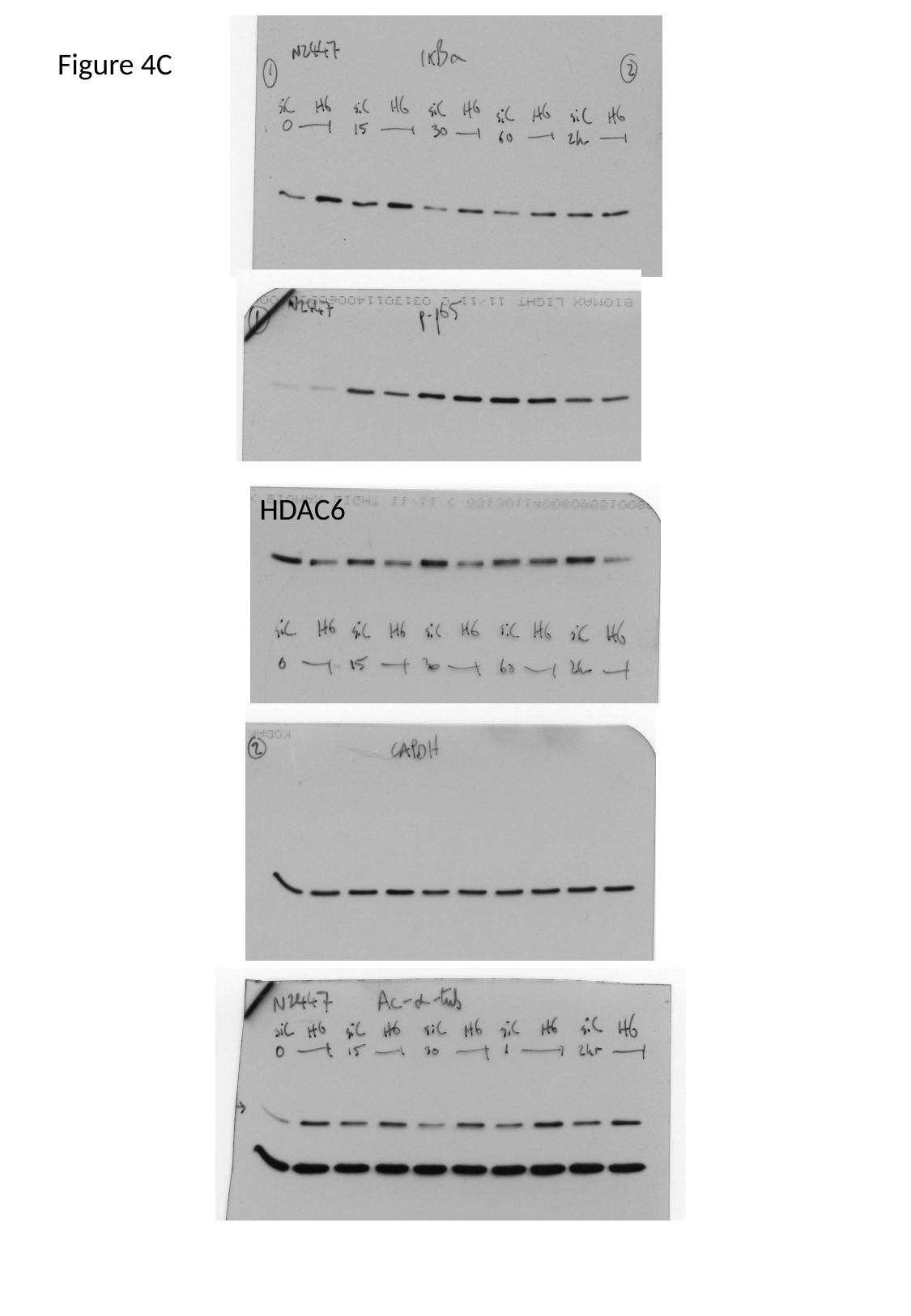

Figure 4C
HDAC6

## Slide 5
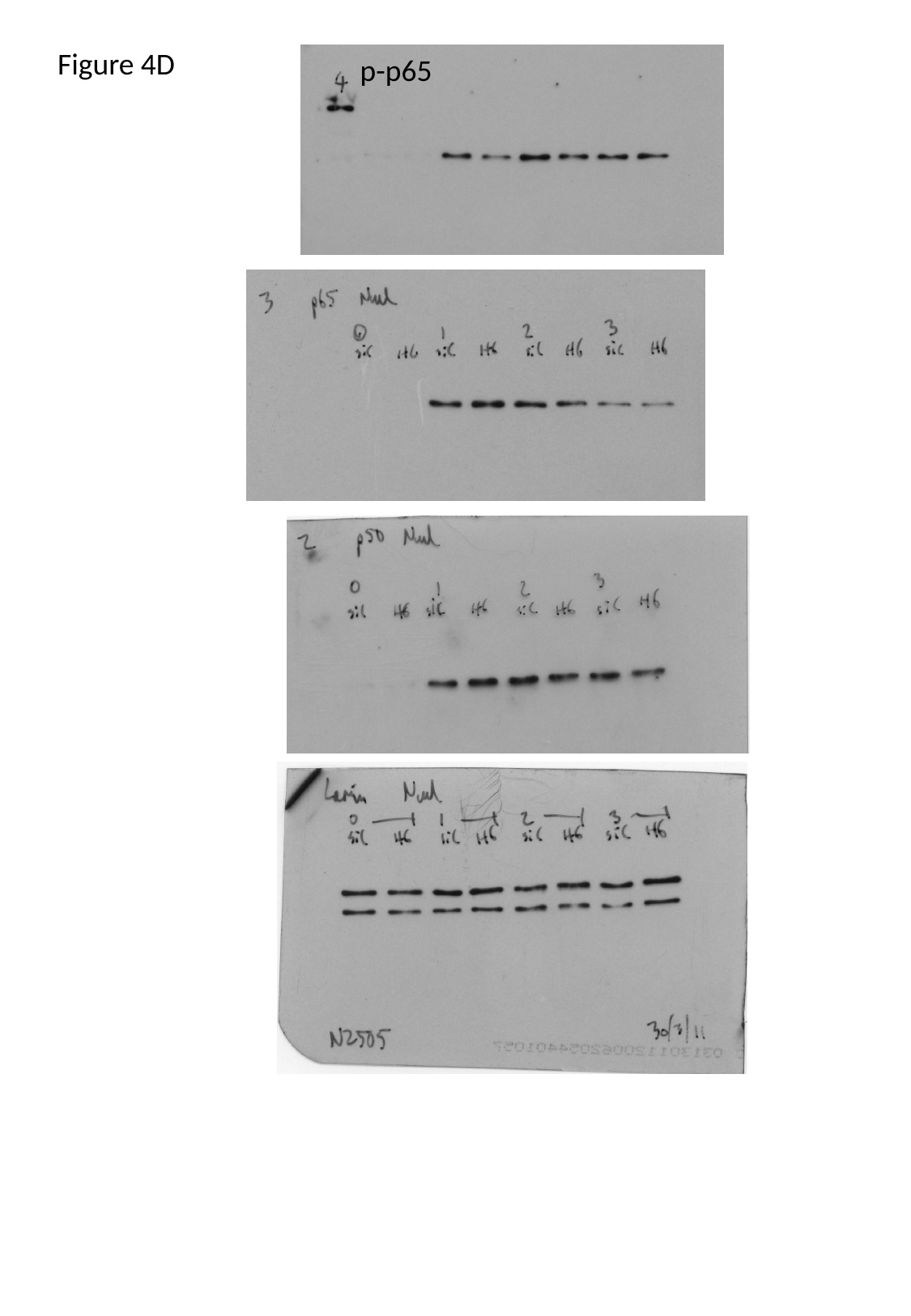

Figure 4D
p-p65

## Slide 6
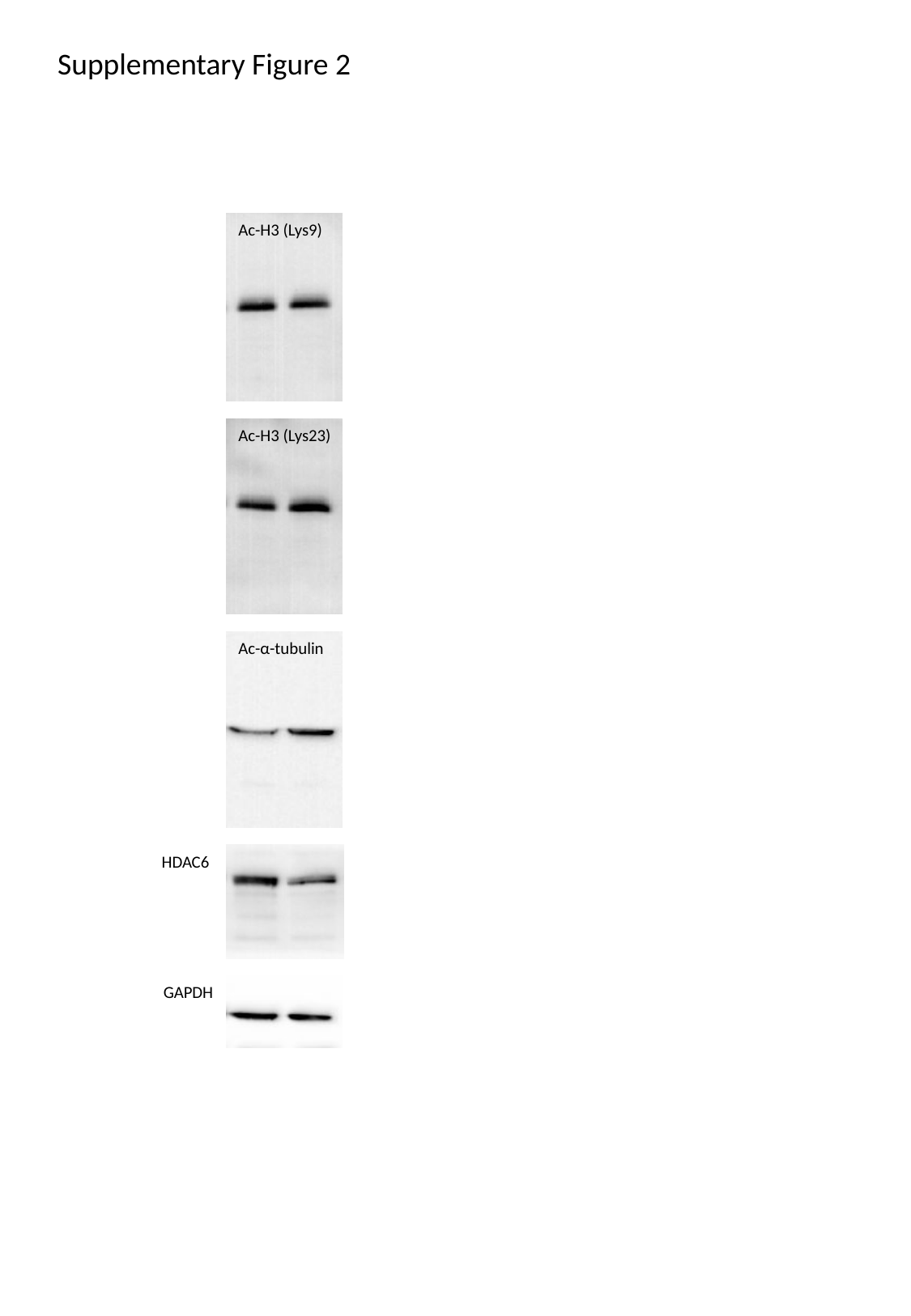

Supplementary Figure 2
Ac-H3 (Lys9)
Ac-H3 (Lys23)
Ac-α-tubulin
HDAC6
GAPDH
