# Supplementary material for: HDAC6 regulates NF-κB signalling to control chondrocyte IL-1-induced MMP and inflammatory gene expression
Source: Sci Rep. 2022 Apr 22;12:6640. doi: 10.1038/s41598-022-10518-z (PMC9033835; doi:10.1038/s41598-022-10518-z)
Supplement: Supplementary file 2 — Supplementary Figures. [file 41598_2022_10518_MOESM2_ESM.docx]

**Supplementary File Legends**

**Supplementary Figure 1. HDAC knockdown efficiency**

SW1353 cells were transfected with the indicated HDAC-targetting siRNAs for 24 hours prior to stimulation with IL-1 for 8 hours. Data are presented as % knockdown compared to non-targetting control siRNA and represent mean ± S.D. Data are representative of a minimum of three independent experiments performed in quadruplicate.

**Supplementary Figure 2. Effect of HDAC6 knockdown and TSA on histone acetylation**

A. SW1353 cells were (A) transfected with the indicated HDAC6-targetting or non-targeting siRNA for 24 hours, or (B) treated with TSA (100ng/ml) for 6 hours. Total protein was extracted and the abundance of the indicated proteins detected by immunoblotting. HDAC6 levels were measured to confirm knockdown and GAPDH was used to confirm equal protein loading.

**Supplementary Figure 3. Toxicity of different concentrations of TSA in SW1353 cells**

SW1353 cells were incubated for 30min with increasing concentrations of TSA before IL-1α (0.5ng/ml) stimulation for 6h was performed. The media was then collected and a toxicity assay was performed using the CytoTox-Glo Cytotoxicity Assay according to manufacturer’s instructions. The luminescence for each sample was detected by GloMax Discover System. For statistical analysis, one-way ANOVA with a Bonferroni multiple comparison test was performed.

**Supplementary Table 1. Primer sequences**

**Supplementary Dataset 1. Microarray analysis of the effect of HDAC6 knockdown on IL-1-induced gene expression in SW1353 chondrocytes**

Gene expression values from microarray analysis of SW1353 cells transfected with QIAGEN HDAC6 siRNA or non-targetting Allstars control siRNA 24 hours prior to stimulation with IL-1 for 6 hours. Log fold change values are presented for contrasts between siHDAC6 and siCon in IL-1 treated cells, and IL-1 treated cells versus control cells.

**Supplementary Dataset 2.** **Microarray analysis of the effect of HDAC inhibitor TSA on IL-1-induced gene expression in SW1353 chondrocytes**

Gene expression values from microarray analysis of SW1353 cells treated with TSA (250ng/ml) or DMSO control for 1 hour prior to stimulation with IL-1α for 8 hours. Log fold change values are presented for contrasts between TSA and DMSO control in IL-1 treated cells, and IL-1 treated cells versus control cells.

**Supplementary Info File 1. Uncropped/full length blots**
