# Supplementary material for: HDAC6 regulates NF-κB signalling to control chondrocyte IL-1-induced MMP and inflammatory gene expression
Source: Sci Rep. 2022 Apr 22;12:6640. doi: 10.1038/s41598-022-10518-z (PMC9033835; doi:10.1038/s41598-022-10518-z)
Supplement: Supplementary file 3 — Supplementary Figure 1. [file 41598_2022_10518_MOESM3_ESM.pptx]

## Slide 1
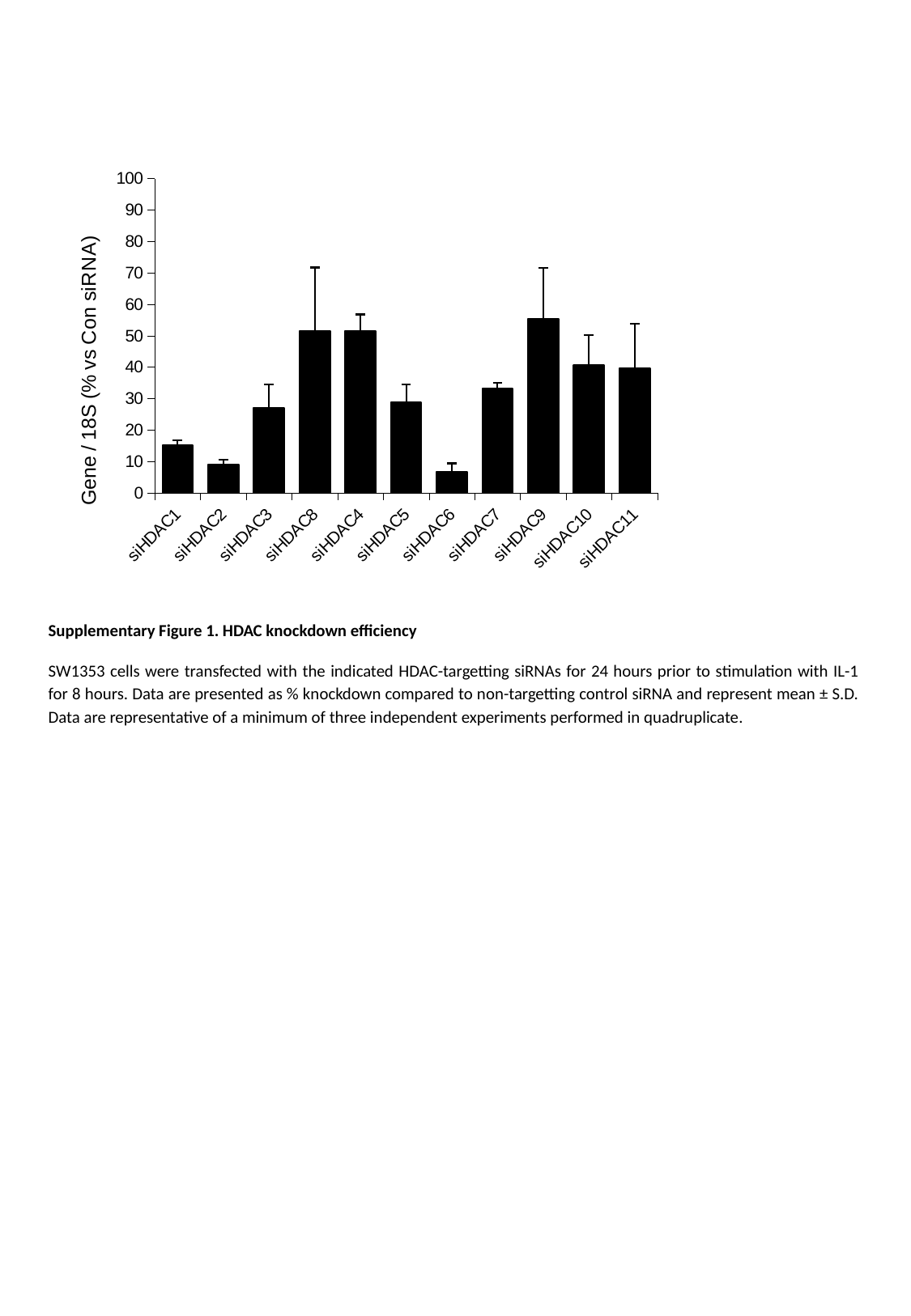

### Chart
| Category | |
|---|---|
| siHDAC1 | 15.104317824241967 |
| siHDAC2 | 8.909226267289373 |
| siHDAC3 | 27.087222544928906 |
| siHDAC8 | 51.638552458745885 |
| siHDAC4 | 51.49155508529378 |
| siHDAC5 | 28.785685237261482 |
| siHDAC6 | 6.754502468109196 |
| siHDAC7 | 33.2515798701458 |
| siHDAC9 | 55.424589896430575 |
| siHDAC10 | 40.58053915417458 |
| siHDAC11 | 39.59614866732707 |Supplementary Figure 1. HDAC knockdown efficiency
SW1353 cells were transfected with the indicated HDAC-targetting siRNAs for 24 hours prior to stimulation with IL-1 for 8 hours. Data are presented as % knockdown compared to non-targetting control siRNA and represent mean ± S.D. Data are representative of a minimum of three independent experiments performed in quadruplicate.
