# Supplementary material for: HDAC6 regulates NF-κB signalling to control chondrocyte IL-1-induced MMP and inflammatory gene expression
Source: Sci Rep. 2022 Apr 22;12:6640. doi: 10.1038/s41598-022-10518-z (PMC9033835; doi:10.1038/s41598-022-10518-z)
Supplement: Supplementary file 4 — Supplementary Figure 2. [file 41598_2022_10518_MOESM4_ESM.pptx]

## Slide 1
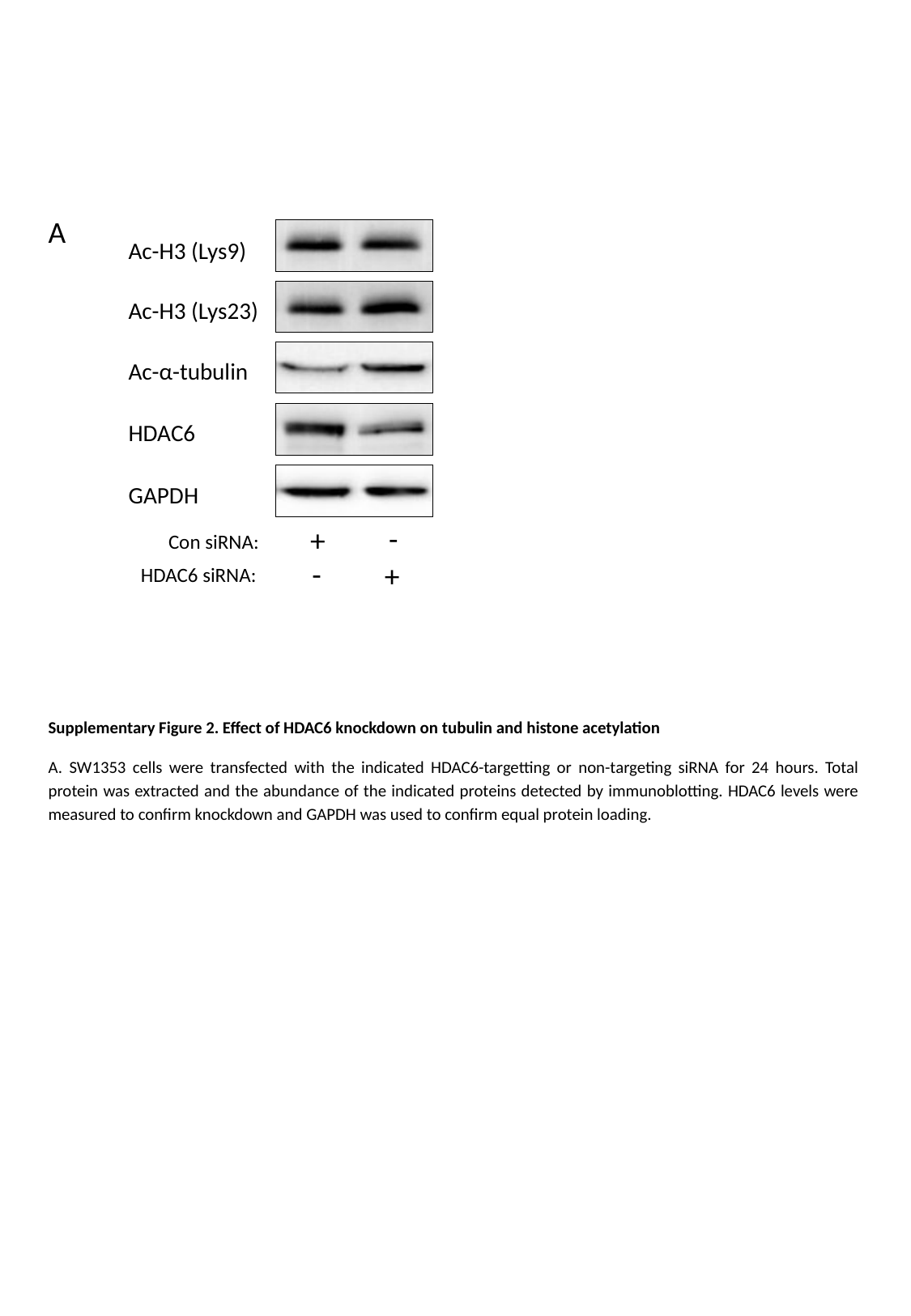

A
Ac-H3 (Lys9)
Ac-H3 (Lys23)
Ac-α-tubulin
HDAC6
GAPDH
-
+
Con siRNA:
-
+
HDAC6 siRNA:
Supplementary Figure 2. Effect of HDAC6 knockdown on tubulin and histone acetylation
A. SW1353 cells were transfected with the indicated HDAC6-targetting or non-targeting siRNA for 24 hours. Total protein was extracted and the abundance of the indicated proteins detected by immunoblotting. HDAC6 levels were measured to confirm knockdown and GAPDH was used to confirm equal protein loading.
