# Supplementary material for: HDAC6 regulates NF-κB signalling to control chondrocyte IL-1-induced MMP and inflammatory gene expression
Source: Sci Rep. 2022 Apr 22;12:6640. doi: 10.1038/s41598-022-10518-z (PMC9033835; doi:10.1038/s41598-022-10518-z)
Supplement: Supplementary file 5 — Supplementary Figure 3. [file 41598_2022_10518_MOESM5_ESM.pptx]

## Slide 1
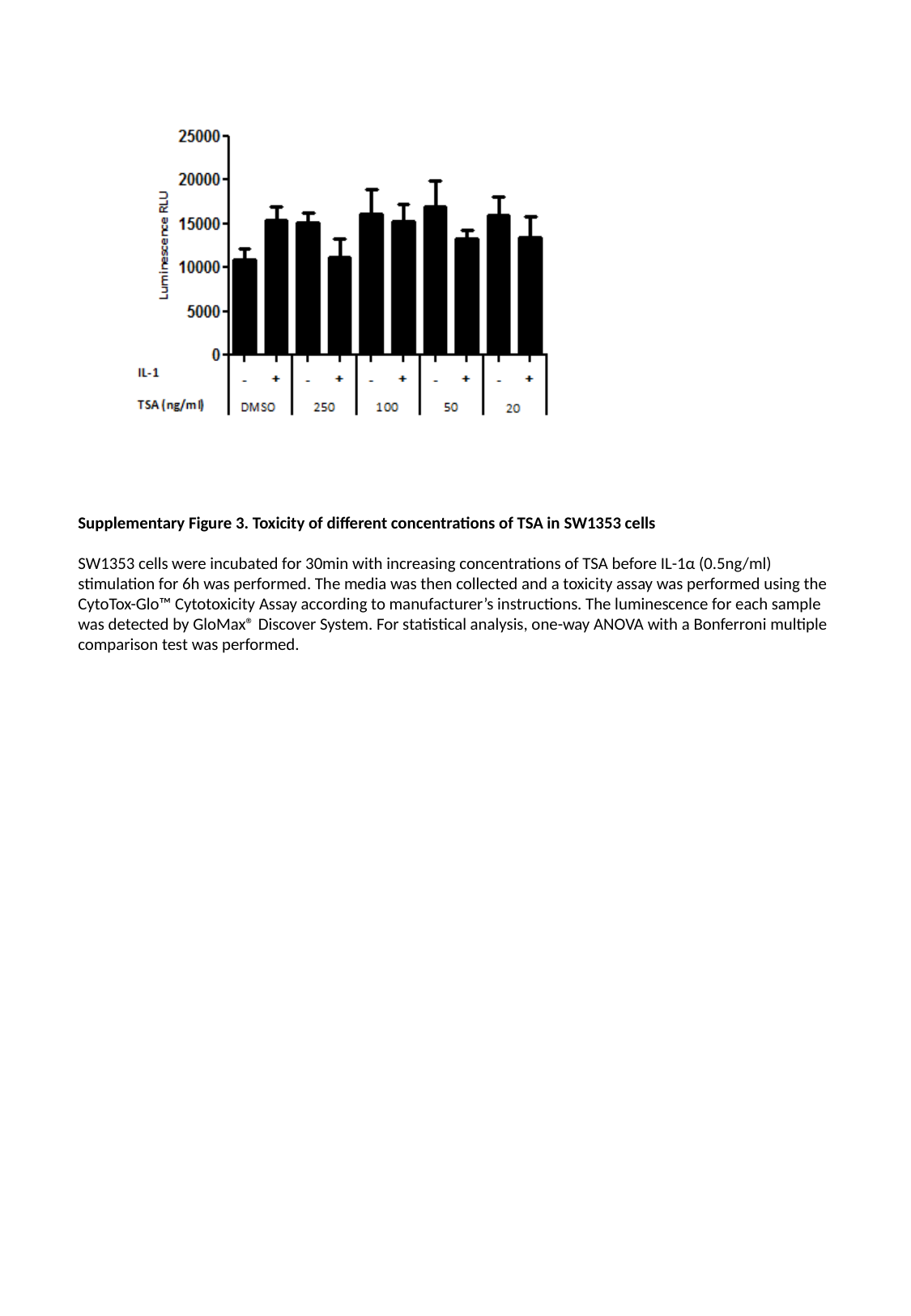

Supplementary Figure 3. Toxicity of different concentrations of TSA in SW1353 cells
SW1353 cells were incubated for 30min with increasing concentrations of TSA before IL-1α (0.5ng/ml) stimulation for 6h was performed. The media was then collected and a toxicity assay was performed using the CytoTox-Glo™ Cytotoxicity Assay according to manufacturer’s instructions. The luminescence for each sample was detected by GloMax® Discover System. For statistical analysis, one-way ANOVA with a Bonferroni multiple comparison test was performed.
